# Inhalation of Ultrafine Particles Alters Blood Leukocyte Expression of Adhesion Molecules in Humans

**DOI:** 10.1289/ehp.7962

**Published:** 2005-09-20

**Authors:** Mark W. Frampton, Judith C. Stewart, Günter Oberdörster, Paul E. Morrow, David Chalupa, Anthony P. Pietropaoli, Lauren M. Frasier, Donna M. Speers, Christopher Cox, Li-Shan Huang, Mark J. Utell

**Affiliations:** 1 Department of Medicine, and; 2 Department of Environmental Medicine, University of Rochester School of Medicine, Rochester, New York, USA; 3 Department of Biostatistics, Bloomberg School of Public Health, Johns Hopkins University, Baltimore, Maryland, USA; 4 Department of Biostatistics, University of Rochester School of Medicine, Rochester, New York, USA

**Keywords:** blood leukocytes, human, monocytes, ultrafine particles

## Abstract

Ultrafine particles (UFPs; aerodynamic diameter < 100 nm) may contribute to the respiratory and cardiovascular morbidity and mortality associated with particulate air pollution. We tested the hypothesis that inhalation of carbon UFPs has vascular effects in healthy and asthmatic subjects, detectable as alterations in blood leukocyte expression of adhesion molecules. Healthy subjects inhaled filtered air and freshly generated elemental carbon particles (count median diameter ~ 25 nm, geometric standard deviation ~ 1.6), for 2 hr, in three separate protocols: 10 μg/m^3^ at rest, 10 and 25 μg/m^3^ with exercise, and 50 μg/m^3^ with exercise. In a fourth protocol, subjects with asthma inhaled air and 10 μg/m^3^ UFPs with exercise. Peripheral venous blood was obtained before and at intervals after exposure, and leukocyte expression of surface markers was quantitated using multiparameter flow cytometry. In healthy subjects, particle exposure with exercise reduced expression of adhesion molecules CD54 and CD18 on monocytes and CD18 and CD49d on granulocytes. There were also concentration-related reductions in blood monocytes, basophils, and eosinophils and increased lymphocyte expression of the activation marker CD25. In subjects with asthma, exposure with exercise to 10 μg/m^3^ UFPs reduced expression of CD11b on monocytes and eosinophils and CD54 on granulocytes. Particle exposure also reduced the percentage of CD4^+^ T cells, basophils, and eosinophils. Inhalation of elemental carbon UFPs alters peripheral blood leukocyte distribution and expression of adhesion molecules, in a pattern consistent with increased retention of leukocytes in the pulmonary vascular bed.

Exposure to particulate matter (PM) air pollution is associated with increased respiratory and cardiovascular morbidity and mortality (Peters et al. 2000, 2001a; Pope et al. 2004). Plausible mechanisms explaining the cardiovascular effects of particle exposure have not been clearly defined (Utell et al. 2002). However, recent studies provide evidence that PM exposure is associated with systemic inflammation and changes in vascular function that have been implicated in the pathophysiology of cardiovascular disease, providing clues to possible mechanisms. PM exposure has been associated with increased systolic blood pressure (Ibald-Mulli et al. 2001), plasma viscosity (Peters et al. 1997a), C-reactive protein (Peters et al. 2001b), fibrinogen (Pekkanen et al. 2000), and release of leukocytes from the bone marrow (Mukae et al. 2001; Tan et al. 2000). Increases in ambient concentrations of PM were associated with increased blood leukocyte and platelet counts, as well as fibrinogen (Schwartz 2001). Brook et al. (2002) found evidence for systemic vasoconstriction in resting human subjects exposed to concentrated ambient air particles and ozone.

Ultrafine particles (UFPs), defined as particles with a diameter < 100 nm, have been hypothesized as contributors to cardiovascular effects of PM (Seaton et al. 1995) because, compared with fine particles at similar mass concentrations, they have greater pulmonary deposition efficiency (Chalupa et al. 2004; Daigle et al. 2003), induce more pulmonary inflammation (Li et al. 1999; Oberdörster et al. 1995), have enhanced oxidant capacity (Brown et al. 2001; Li et al. 2003), have a higher propensity to penetrate the epithelium and reach interstitial sites (Stearns et al. 1994), and may even enter the systemic circulation in humans (Nemmar et al. 2002; Oberdörster et al. 2002).

Relatively few epidemiologic studies have examined the health effects of UFP exposure because most ambient air monitoring measures particle mass, and there is relatively poor correlation between particle mass (dominated by fine particles) and particle number (dominated by UFPs). However, a recent study in Erfurt, Germany, found associations between ambient UFPs and mortality (Wichmann et al. 2000). In a study of patients with stable coronary artery disease (Pekkanen et al. 2002), investigators performed repeated exercise tests concurrent with monitoring of ambient particle mass and number counts. Significant independent effects were found for both fine particles and UFPs on the degree of ST-segment depression on the electrocardiogram during exercise.

Asthma, a disease characterized by airway inflammation, confers an increased risk for PM health effects (Atkinson et al. 2001; Lipsett et al. 1997; Tolbert et al. 2000). There is evidence for activation of lung leukocytes and pulmonary vascular endothelium in subjects with asthma, particularly during exacerbations (Ohkawara et al. 1995). Activation of T-lymphocytes with production of “type 2” inflammatory cytokines drives the recruitment and retention of eosinophils in the airway, which contribute to the chronic epithelial injury characteristic of this disease (Corrigan and Kay 1990; Wilson et al. 1992). Treatment with inhaled corticosteroids reduces expression of activation markers CD25 and human leukocyte antigen (HLA)-DR in lung lymphocytes and also reduces HLA-DR expression in blood lymphocytes (Wilson et al. 1994). In asthma, blood CD4^+^ T cells express increased mRNA for interleukin (IL)-4, IL-5, and granulocyte macrophage colony stimulating factor, and IL-5 mRNA expression correlates with asthma severity and eosinophilia (Corrigan et al. 1995). Allergen challenge in subjects with asthma causes a reduction in blood CD4^+^ T cells (Walker et al. 1992) and an increase in airway CD4^+^ cells (Virchow et al. 1995). UFP exposure may worsen asthma by further shifting lymphocyte responses to the type 2 phenotype, by further activating resident lymphocytes, by increasing the likelihood that lymphocytes will encounter antigen, and/or by increasing penetration of allergen through an injured epithelium.

We have initiated controlled exposure studies with carbon UFPs in humans, as a surrogate for environmental UFPs, demonstrating that UFPs have a high pulmonary deposition efficiency in healthy subjects (Daigle et al. 2003), which is further increased in subjects with asthma (Chalupa et al. 2004). Exposure to 50 μg/m^3^ carbon UFPs caused a reduction in the pulmonary diffusing capacity for carbon monoxide (Pietropaoli et al. 2004b) associated with reductions in the systemic vascular response to increased flow (Pietropaoli et al. 2004a), without significant effects on symptoms, airway inflammation, lung function, or markers of blood coagulation (Pietropaoli et al. 2004c). We hypothesized that inhalation of UFPs alters vascular function, detectable as alterations in blood leukocyte distribution, activation, and expression of adhesion molecules. We further hypothesized that people with asthma, who have airway and systemic inflammation at baseline as well as enhanced UFP deposition, have enhanced susceptibility to these vascular effects. In this article we present detailed analyses of venous blood leukocytes from subjects participating in four separate studies involving carbon UFP exposure: three protocols with varying exposure concentrations in healthy subjects, and one protocol with asthmatic subjects. Some data in this article have been presented previously in abstract form (Frampton et al. 2004).

## Materials and Methods

### Subjects.

Written, informed consent was obtained from all subjects, and the studies were approved by the Research Subjects Review Board of the University of Rochester. Fifty-six never-smoking subjects 18–40 years of age (40 healthy and 16 with asthma) participated and were paid a stipend. Subjects were not studied within 6 weeks of a respiratory infection. Healthy subjects were required to have normal spirometry, a normal 12-lead electrocardiogram, and no history of chronic respiratory disease.

Inclusion criteria for subjects with asthma have been reported previously (Chalupa et al. 2004). These criteria included a consistent clinical history, and either a significant bronchodilator response or airway hyper-responsiveness to methacholine. The severity was consistent with mild intermittent to moderate persistent asthma (National Institutes of Health 1997). Subjects with forced expiratory volume in 1 sec (FEV_1_) < 70% of predicted at baseline screening, or with > 20% reduction in FEV_1_ after the screening exercise, were excluded.

### Study design.

Each study used a crossover design in which each subject was exposed to filtered air and to UFPs, so that each subject served as his or her own control. Within each study, the order of air/UFP exposure was randomized, and the randomization was blocked by order of presentation and sex, so that equal numbers of men and women inhaled air first or UFPs first. Exposures were blinded to both subjects and investigators.

Table 1 provides details of each study protocol. The first, UPREST, involved 12 (six female) subjects exposed at rest to approximately 10 μg/m^3^ UFPs or filtered air for 2 hr. The second study protocol, UPDOSE, involved 12 subjects (six female) with three 2-hr exposures with exercise for each subject: approximately 10 μg/m^3^ UFPs, approximately 25 μg/m^3^ UFPs, and filtered air. Subjects exercised on a bicycle ergometer for 15 min of each half hour at an intensity adjusted to increase the minute ventilation to approximately 20 L/min/m^2^ body surface area. For safety reasons, the order of exposure was randomized in a restricted fashion, so that each subject received the 10-μg/m^3^ exposure before the 25-μg/m^3^. The third protocol, UP50, involved 16 healthy subjects (eight female) exposed to approximately 50 μg/m^3^ UFPs and air for 2 hr, with intermittent exercise as in the UPDOSE protocol. The final protocol, UPASTHMA, involved 16 subjects with asthma (eight female) exposed to approximately 10 μg/m^3^ UFPs and air for 2 hr, with intermittent exercise as in the UPDOSE protocol. All exposures were separated by at least 2 weeks.

Exposures to either filtered air or UFPs were administered by mouthpiece (with nose clip) for 2 hr, interrupted by a 10-min break after the first hour. Before and at 0, 3.5, and 21 hr after exposure, blood pressure, heart rate, and oxygen saturation by pulse oximetry were measured, and blood was drawn from an antecubital vein. For UP50 and UPASTHMA, measurements were also obtained 45 hr after exposure.

### Exposure system.

The rationale and design of the exposure facility have been described in detail elsewhere (Chalupa et al. 2002). Briefly, particles [count median diameter ~ 25 nm, geometric standard deviation ~ 1.6] were generated in an argon atmosphere using an electric spark discharge between two graphite electrodes, and then deionized and diluted with filtered air to the desired concentration. Particle number, mass, and size distribution were monitored on both the inspiratory and expiratory sides of the subject. Electronic integration of a pneumotachograph signal provided tidal volume, respiratory frequency, and minute ventilation measurements. Air for the control exposures, and for dilution of the particles, was passed through charcoal and high-efficiency particle filters and was essentially free of particles (0–10 particles/cm^3^).

### Blood leukocyte immunofluorescence analysis.

Fresh heparinized whole blood was stained with three monoclonal antibodies: the marker of interest (Table 2) conjugated to fluorescein isothiocyanate, CD14 conjugated to phycoerythrin, and CD45 conjugated to pericidin chlorophyll protein. This permitted determination of the relative expression of adhesion molecules and other markers separately on polymorphonuclear leukocytes (PMNs), eosinophils, lymphocytes, and monocytes. The appropriate isotype control antibodies were run with each experiment to assist in appropriate gate setting. The adhesion markers shown in Table 2 were measured in each of the study protocols, except for CD18, which was measured in UP50 and UPASTHMA only.

Red blood cells were lysed and cells were analyzed on a FACScan flow cytometer (BD Bioscience, San Jose, CA) equipped with a 15-mW argon ion laser emitting at 488 nm. Ten thousand events were collected from each sample in list mode. Standardized fluorescent microbeads (Quantium 24P and 25P; Bangs Laboratories, Fishers, IN) were run with each experiment to convert mean channel numbers to molecules of equivalent soluble fluorochrome (MESF) (Gavras et al. 1994). This provided a correction for minor day-to-day instrument variations in fluorescence detection.

Total and differential blood leukocyte and platelet counts were performed in the clinical laboratories of Strong Memorial Hospital, using an automated analyzer (Celldyne 4000; Abott Laboratories, Santa Clara, CA).

### Data handling and statistical methods.

Data were entered on a desktop computer using Microsoft Excel and analyzed using SAS (SAS Institute Inc., Cary, NC).

UPREST, UPASTHMA, and UP50 used a standard, two-period crossover design in which each subject received both particles and air. Equal numbers of males and females were included. The order of presentation was randomized separately for each sex, with half of each group of subjects receiving each of the two possible orders. UPDOSE used a three-period crossover design in which each subject received air and both 10-μg/m^3^ and 25-μg/m^3^ concentrations of particles. There were then three possible exposure sequences, depending on where in the sequence the air exposure was placed. Equal numbers of subjects were randomly assigned to each sequence.

Repeated-measures analysis of variance (ANOVA) was used (Wallenstein and Fisher 1977), with order of presentation as a between-subjects factor, with exposure and time as within-subject factors. The analysis included tests for period and carryover effects, although the latter were expected to be minimal because of the nature of the exposures and the length of the washout period. In cases where carryover effects were significant, first-period data were examined separately (Jones and Kenward 1989). Each ANOVA included an examination of residuals as a check on the required assumptions of normally distributed errors with constant variance. If these assumptions were not satisfied, data transformations (e.g., square-root transformation for cell counts) were considered. A *p*-value of 0.05 was required for statistical significance. Data are shown as mean ± SE, unless otherwise indicated.

## Results

### Exposure data and subject characteristics.

Table 3 shows the exposure parameters and subject characteristics for each protocol. Most of the subjects with asthma were atopic (15 of 16), and most (11 of 16) were not on inhaled steroids, long-acting bronchodilators, or leukotriene inhibitors. All subjects completed every exposure; men and women did not differ in the achieved minute ventilation, adjusted for body surface area. There were no significant effects of UFP exposure on ventilatory parameters or pulmonary function; these results, and UFP deposition, have been published previously (Daigle et al. 2003).

The UPREST protocol, with exposures at rest to 10 μg/m^3^ UFPs, showed no convincing differences between particle and air exposure for leukocyte expression of adhesion molecules or total and differential leukocyte counts. There were rare statistically significant comparisons, but the significance levels were modest, and the data did not suggest a consistent biologic response. Overall, exposure to 10 μg/m^3^ UFPs at rest had no significant effects on blood leukocytes.

Findings from the three studies involving exercise are described below.

### Blood leukocyte expression of adhesion molecules.

In these studies, quantitative surface expression of molecules that mediate leukocyte-endothelial interactions served as an indirect indicator of exposure effects on pulmonary vascular endothelial function. The use of flow cytometry with calibrated fluorescent beads allowed quantitation of small changes in surface marker density. Adhesion molecule expression for monocytes and PMNs in the three protocols involving exercise is shown in Tables 4–6.

### UPDOSE.

**Figure 1:**
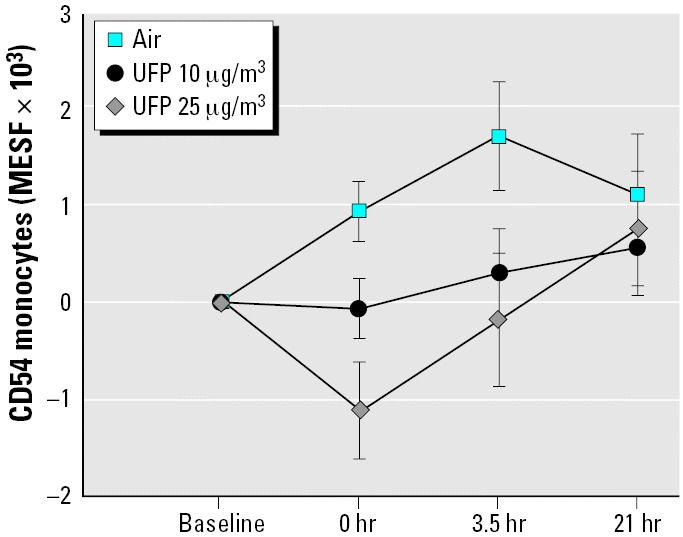
Changes in monocyte expression of CD54 (ICAM-1), UPDOSE protocol. In this and following figures, data are shown as mean ± SE changes from baseline. Nominal UFP exposure concentrations are shown in μg/m^3^. Exposure, *p* = 0.012.

UFP exposure caused a concentration-related reduction in monocyte expression of CD54 [intercellular adhesion molecule-1 (ICAM-1) (exposure effect, *p* = 0.0012); Figure 1]. Expression increased after exposure to filtered air and decreased with 25 μg/m^3^ UFPs, with differences resolving by 21 hr after exposure. Expression of CD62L showed a significant exposure–sex interaction (*p* = 0.0006; data not shown), with expression increasing in females but decreasing in males relative to air exposure. However, these findings lacked a clear concentration response.

### UP50.

**Figure 2:**
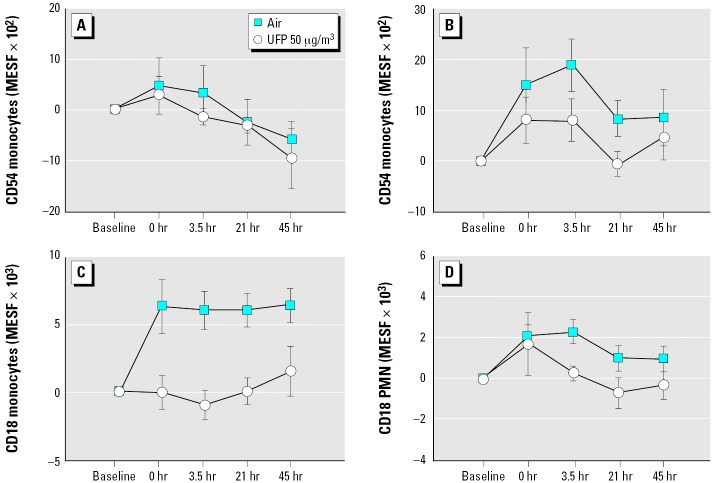
Changes in leukocyte expression of adhesion molecules, UP50 protocol. (*A*) Monocyte expression of CD54, females. UFP × sex, *p* = 0.025. (*B*) Monocyte expression of CD54, males. UFP × sex, *p* = 0.025. (*C*) Monocyte expression of CD18. UFP, *p* = 0.0002. (*D*) PMN expression of CD18. UFP, *p* = 0.023.

Exposure to 50 μg/m^3^ UFPs also reduced expression of CD54 on monocytes (Figure 2A,B), but to a greater extent in males (exposure–sex interaction, *p* = 0.025). The percentage of monocytes expressing CD54 was also reduced (*p* = 0.035; data not shown). UFP exposure persistently blunted the air-related increase in CD18 expression on monocytes (*p* = 0.0002; Figure 2C). Expression of CD18 was also reduced on PMNs (Figure 2D), and ANOVA indicated significantly increased CD11a expression on PMNs (exposure–time interaction, *p* = 0.037; data not shown).

### UPASTHMA.

As expected, we found baseline differences between healthy and asthmatic subjects in leukocyte expression of adhesion molecules; these data are shown in Table 7. For example, monocyte expression of CD11b, CD54, and CD62L was higher in subjects with asthma than in healthy subjects.

**Figure 3:**
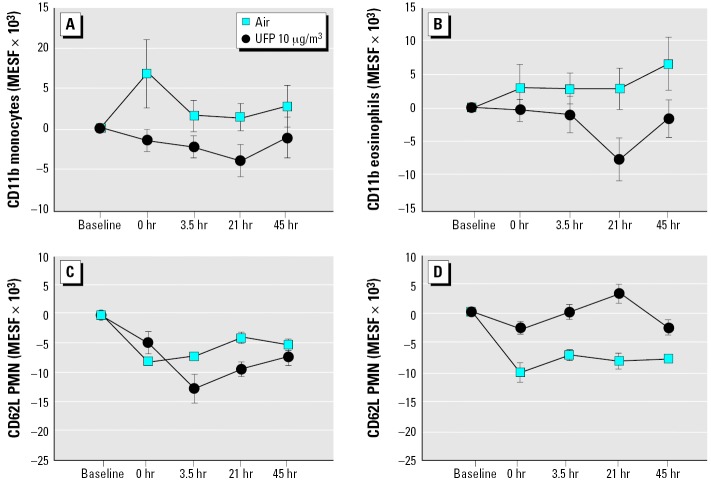
Changes in leukocyte expression of adhesion molecules, UPASTHMA protocol. (*A*) Monocyte expression of CD11b. UFP, *p* = 0.029. (*B*) Eosinophil expression of CD11b. UFP, *p* = 0.015. (*C*) PMN expression of CD62L, females. UFP × sex, *p* = 0.011. (*D*) PMN expression of CD62L, males. UFP × sex, *p* = 0.011.

In subjects with asthma, exposure to 10 μg/m^3^ UFPs reduced expression of CD11b on blood monocytes (*p* = 0.029; Figure 3A) and also reduced expression on eosinophils (*p* = 0.015; Figure 3B). Expression of CD62L on PMNs increased in males but not females (exposure–sex interaction, *p* = 0.011; Figure 3C,D). Expression of CD54 on PMNs decreased, with the greatest difference from control at 45 hr after exposure (exposure–time interaction, *p* = 0.031; data not shown).

### Lymphocyte subsets and activation.

**Figure 4:**
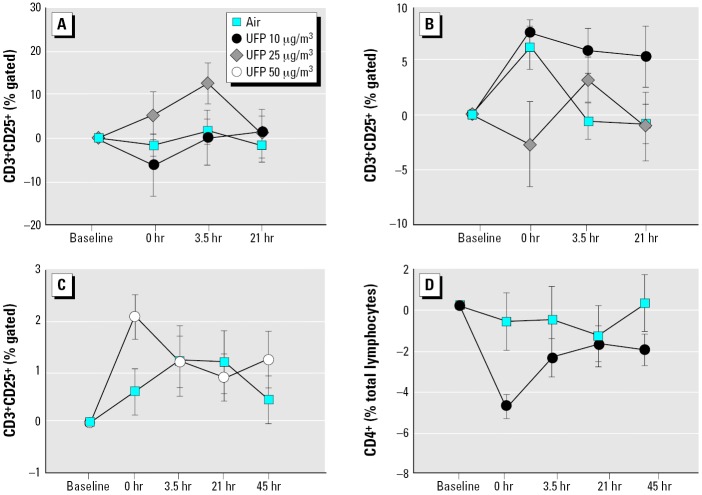
Changes in blood T-lymphocyte subsets. (*A*) UPDOSE protocol, percentage of CD25^+^ cells within the T-cell (CD3^+^) gate, females. UFP × sex, *p* = 0.0024. (*B*) UPDOSE protocol, CD3^+^CD25^+^ T cells, males. UFP × sex, *p* = 0.0024. (*C*) UP50 protocol, CD3^+^CD25^+^ T cells, all subjects. UFP × time, *p* = 0.085. (*D*) UPASTHMA protocol, CD4^+^ T cells, all subjects. UFP × time, *p* = 0.021.

There was evidence for increased activated T cells after UFP exposure in healthy subjects. In UPDOSE, CD25 expression on CD3^+^ T cells increased in females, but not in males, early after exposure to 25 μg/m^3^ UFPs (exposure–sex interaction, *p* = 0.002; Figure 4A,B). In UP50, exposure to 50 μg/m^3^ increased CD25 expression on T cells 0 hr after exposure (*p* = 0.001 by paired *t*-test at 0 hr after exposure; *p* = 0.085 by ANOVA; Figure 4C). There were no other changes in lymphocyte subsets in the healthy subjects.

In UPASTHMA, CD4^+^ T cells decreased immediately after exposure to UFPs, compared with air (exposure–time interaction, *p* = 0.021; Figure 2D). There were no significant effects on other lymphocyte subsets or CD25 expression. However, the percentage of T-lymphocytes expressing the activation marker CD25 was higher in asthmatic subjects than in healthy subjects before exposure (UPASTHMA, 33.0 ± 3.3%, vs. UPDOSE, 27.0 ± 2.5%; *p* = 0.04).

Overall, the data suggest that UFP exposure induces activation (healthy subjects) or sequestration (subjects with asthma) of T-lymphocytes.

### Blood leukocyte and platelet counts.

**Figure 5:**
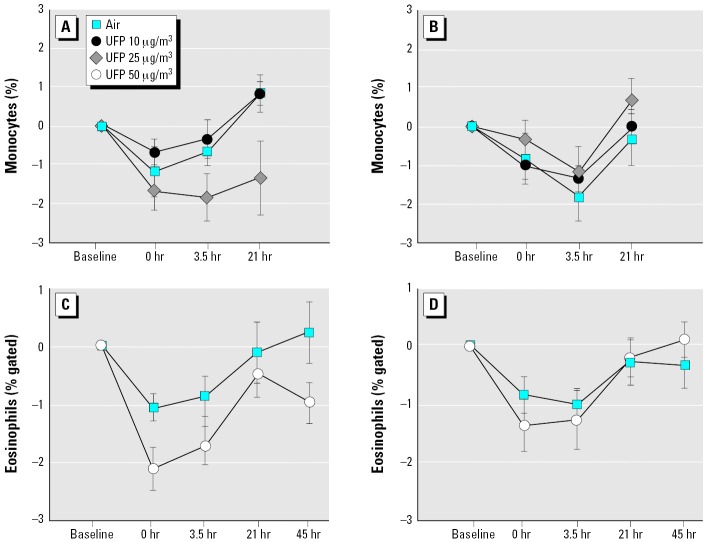
Changes in percentage of blood leukocytes with exposure to UFPs. (*A*) UPDOSE protocol, monocytes, females. UFP × sex, *p* = 0.0015. (*B*) UPDOSE protocol, monocytes, males. UFP × sex, *p* = 0.0015. (*C*) UP50 protocol, eosinophils, females. UFP × time × sex, *p* = 0.01. (*D*) UP50 protocol, eosinophils, males. UFP × time × sex, *p* = 0.01.

In each of the protocols involving exercise (UPDOSE, UP50, and UPASTHMA), consistent postexposure increases were seen in the total leukocyte count and the percentage of PMNs, with decreases in the percentage of eosinophils and monocytes. In the UPDOSE protocol, ANOVA showed a significant exposure–sex interaction for an effect on the percentage of blood monocytes (*p* = 0.0015). As shown in Figure 5A,B, in females monocytes decreased after exposure to 25 μg/m^3^ and did not return to baseline at 21 hr after exposure. This observation was confirmed when monocyte numbers were analyzed by flow cytometry, using light scatter and CD14 expression (overall effect of UFPs, *p* = 0.035; exposure–sex interaction, *p* = 0.002). A significant decrease in blood basophils in females was also seen with both UFP concentrations (exposure–sex interaction, *p* = 0.015; data not shown).

Exposure to 50 μg/m^3^ UFPs caused small reductions in the percentage of eosinophils, with a larger effect in females (Figure 5C,D). There were no significant effects on the percentage of blood monocytes, PMNs, or basophils in this protocol.

In subjects with asthma exposed to 10 μg/m^3^ UFPs, basophils decreased in both men and women at 0 and 3.5 hr after exposure to UFPs, compared with air exposure (exposure–time interaction, *p* = 0.02; data not shown). The percentage of blood eosinophils as determined by flow cytometry decreased 0 and 3.5 hr after exposure, with greater reductions after UFP exposure than after air (*p* = 0.049).

UFP exposure did not change platelet counts in any of the exposure protocols.

These data suggest that exposure to UFPs with exercise causes small changes in blood leukocyte differential counts in both healthy and asthmatic subjects.

## Discussion

The objective of these studies was to determine whether inhalation of carbon UFPs has vascular effects in healthy subjects, and in subjects with asthma. We postulated that changes in blood leukocyte phenotype and expression of adhesion molecules would serve as a “window” on vascular inflammatory effects after inhalation challenge. Although the specific findings differed among the protocols, all three protocols with exercise showed UFP-associated reductions in expression of adhesion molecules on leukocytes, mainly ICAM-1 (CD54) and the β_2_ integrins CD11b and CD18. There were significant differences between healthy and asthmatic subjects in leukocyte expression of adhesion molecules, when measured before exposure (Table 7). For example, blood monocytes from subjects with asthma showed decreased expression of CD11a and increased expression of CD11b, CD49d, and CD54 relative to healthy subjects. This may reflect relative activation or priming of circulating leukocytes as a consequence of airway inflammation. In subjects with asthma, inhalation of UFPs reduced expression of CD11b on monocytes and eosinophils (Figure 3) and reduced CD54 expression on PMNs (Table 6).

In addition, the data suggested subtle reductions relative to air exposure in the percentage of blood monocytes, eosinophils, and basophils. There was evidence for activation of CD4^+^ T-lymphocytes in healthy subjects and transient reductions in CD4^+^ T-cell numbers in asthmatic subjects. Sex interactions were seen for some of these changes. A summary of these findings is shown in Table 8.

The findings provide evidence that inhalation of elemental carbon UFPs, with intermittent exercise, causes phenotypic alterations in blood leukocytes at concentrations as low as approximately 10 μg/m^3^ or approximately 2 × 10^6^ particles/cm^3^. However, the overall nature and direction of the changes do not suggest increased systemic inflammation. This is consistent with the lack of evidence for airway or systemic inflammation that we have reported previously for these studies (Pietropaoli et al. 2004a, 2004c).

The reductions in leukocyte subsets and adhesion molecule expression seen in these studies suggest the possibility of leukocyte sequestration or margination in response to UFP exposure. The relative reductions in monocyte, basophil, and eosinophil percentages may result from slightly prolonged transit time through the pulmonary circulation after exposure to UFPs, possibly as a consequence of pulmonary vasoconstriction. The reductions in expression of the adhesion molecules CD54, CD11b, and CD18 are consistent with this hypothesis. Blood leukocytes normally marginate in the lung, requiring several seconds to transit the pulmonary circulation (Doerschuk 2003). PMNs are larger than pulmonary capillaries and must deform in order to transit. The integrins CD11a and CD11b are expressed as dimers with CD18 and mediate blood leukocyte recruitment to areas of inflammation and injury via specific receptors on vascular endothelial cells. Activation of monocytes and PMNs increases expression of CD11b and CD18 and decreases cell deformability through actin polymerization (Anderson et al. 2001), slowing transit time. Exercise increases pulmonary blood flow and decreases leukocyte transit time through the pulmonary circulation, leading to mobilization of the pulmonary leukocyte pool into the systemic vascular pool. Van Eeden et al. (1999) have shown that maximal exercise increases the blood leukocyte count and also increases expression of CD11b on peripheral blood PMNs, suggesting that cells expressing higher levels of CD11b preferentially marginate in the pulmonary circulation and are “flushed out” with exercise. Thus, our data are consistent with, but do not prove, increased retention of leukocytes expressing higher levels of adhesion molecules in the pulmonary vascular bed in response to UFP exposure.

Pulmonary vasoconstriction in response to UFP exposure would be expected to delay leukocyte transit through the lung. We have reported (Pietropaoli et al. 2004b) that, in the UP50 protocol, UFP exposure caused reductions in the diffusing capacity for carbon monoxide, without effects on the forced vital capacity, consistent with reduced vascular perfusion or reduced ventilation/perfusion matching. We also reported preliminary findings (Pietropaoli et al. 2004a) of subtle but significant effects on systemic flow-mediated vascular dilatation, and a decrease in blood nitrate levels, suggesting the vascular effects may result from decreased nitric oxide availability. Batalha et al. (2002) have shown pulmonary vaso-constriction in rats exposed to concentrated ambient fine particles.

Alternative mechanisms for reductions in leukocyte and their surface markers include *a*) direct effects of UFPs on blood leukocytes, reducing surface marker expression through shedding, redistribution, or internalization; *b*) indirect effects of mediators released by vascular endothelium, such as nitric oxide, which has anti-inflammatory properties (Lefer 1997), reduces endothelial expression of adhesion molecules via inhibition of nuclear factor κB activation, and reduces monocyte adhesion to endothelium (De Caterina et al. 1995); *c*) adsorption of soluble cytokines, such as transforming growth factor-β, onto the surface of the particles, reducing inflammatory effects (Kim et al. 2003); *d*) recruitment of immature leukocytes from the bone marrow in response to UFP inhalation, as has been suggested in previous studies of fine particle exposure (Tan et al. 2000); and *e*) selective toxicity of UFPs for activated blood leukocytes, inducing apoptosis of specific cell subsets.

The two protocols with exercise in healthy subjects showed increased expression of CD25 on blood T-lymphocytes, and subjects with asthma showed a transient reduction in CD4^+^ lymphocytes after UFP exposure. CD25 is the α-chain of the IL-2 receptor; IL-2 promotes lymphocyte proliferation. We found that lymphocyte CD25 expression was higher in subjects with asthma than in healthy subjects, confirming previous observations that people with asthma have a higher percentage of circulating activated T-lymphocytes (Corrigan and Kay 1990), which may explain why UFP exposure did not increase it further in these subjects. The rapid and transient nature of the reduction in CD4^+^ T cells suggests redistribution or margination of cells, as postulated above for other blood leukocytes.

The changes in response to carbon UFP exposure reported in these studies were generally small and would not be expected to adversely affect healthy and mildly asthmatic subjects similar to those studied. However, ambient UFPs contain reactive organic species and transition metals that may induce greater effects than those we observed. People with severely compromised cardiovascular status may experience adverse effects from even small changes in vascular homeostasis. Furthermore, prolonged, repeated exposures may hasten the progression of atherosclerosis, as has been suggested in an epidemiology study of fine particle exposure (Künzli et al. 2005).

The UFP number concentrations used in these studies are higher than UFP background concentrations but are relevant to episodic levels seen in specific situations. UFPs are always present in ambient air, with background urban levels in the range of 40,000–50,000 particles/cm^3^ or estimated mass concentrations of 3–4 μg/m^3^ (Peters et al. 1997b). Episodic increases have been documented to 300,000 particles/cm^3^, or estimated to approximately 50 μg/m^3^ UFPs as an hourly average (Brand et al. 1991, 1992). Particle numbers inside a vehicle on a major highway reached 10^7^ particles/cm^3^ (Kittelson et al. 2001), comparable with the highest concentrations used in our studies.

Although not specifically powered to detect sex differences, these studies were designed to include an analysis of sex interactions with the effects of UFP exposure. In the UPDOSE protocol, females showed greater decreases in blood monocytes (Figure 5A) and basophils and greater increases lymphocyte CD25 expression (Figure 4A) compared with males. Females also showed decreased eosinophils in the UP50 protocol (Figure 5C). In UPASTHMA, expression of L-selectin (CD62L) on PMNs was increased in males (Figure 3B). It is possible that males and females differ in their cardiovascular responses to particle exposure. There are known sex differences in leukocyte function and cardiovascular responses, based in part on hormonal influences. For example, females have a higher percentage of CD4^+^ T cells and a higher CD4^+^:CD8^+^ ratio than do males. Stimulated blood monocytes from females produce more prostaglandin E_2_ (Leslie and Dubey 1994) and less tumor necrosis factor-α and IL-6 (Schwarz et al. 2000) than those from males. There are also sex differences in endothelial function and antioxidant defenses that may affect vascular response to inhaled challenge. However, we do not feel that these studies have convincingly established or excluded significant sex differences in responses to carbon UFPs.

There are limitations to this study. First, our particles were laboratory-generated elemental carbon, without significant organic species, metals, oxides, nitrates, or sulfates. The findings of these studies may not be representative of exposure to ambient particles, which are a mix of ultrafine, fine, and coarse particles, with reactive organic species, metals, and oxidants in addition to elemental carbon. These and other chemical species may enhance pulmonary and vascular effects. Second, each protocol involved a fairly large number of measurements, and some statistically significant changes may have been chance related. Our approach was to consider results that showed consistency within and across protocols and to discount findings of isolated statistical significance that were not supported by other data. The observations of UFP effects on leukocyte distribution and surface marker expression meet those criteria.

## Conclusions

Overall, the findings from these studies provide evidence that inhalation of carbon UFPs, with exercise, reduces peripheral blood monocytes, eosinophils, and basophils and reduces expression of some adhesion molecules on monocytes and PMNs. When considered in light of other evidence, the leukocyte changes may be a consequence of endothelial activation or vasoconstriction in the pulmonary and/or systemic circulation.

## Figures and Tables

**Table 1:** Study design (mean ± SD).

	UPREST	UPDOSE	UP50	UPASTHMA
No. of subjects	12	12	16	16
Subject age (years)	30.1 ± 8.9	26.9 ± 5.8	26.9 ± 6.5	23.0 ± 2.7
FEV_1_ (% predicted)	103.8 ± 8.0	106.3 ± 16.6	102.8 ± 9.5	97.6 ± 5.0
Nominal^a^ particle mass (μg/m^3^)	0, 10	0, 10, 25	0, 50	0, 10
Rest/exercise	Rest	Intermittent exercise	Intermittent exercise	Intermittent exercise

a The target mass concentration of UFPs for each protocol.

**Table 2:** Leukocyte markers measured in each protocol.

Cluster designation	Name	Source (clone)	Description
CD3		BD Bioscience^a^ (SK7)	Marker of T-lymphocytes
CD4		BD Bioscience (SK3)	Marker of T-helper lymphocytes
CD8		BD Bioscience (SK1)	Marker of T-cytotoxic lymphocytes
CD11a	Leukocyte function antigen-1	GenTrak^b^ (38) or Coulter^c^ (25.3.1)	Part of β_2_ integrin adhesion molecule complex
CD11b	Mac-1	Ancell^d^ (ICRF44)	Subunit of complement receptor 3, part of β2 integrin adhesion molecule complex
CD18^e^		Pharmigen^a^ (6.7) or BD Bioscience (L130)	Part of β_2_ adhesion molecule complex with CD11a and CD11b
CD25	Tac	BD Bioscience (2A3)	Epitope of IL-2 receptor, activation marker on lymphocytes
CD49d	Very late antigen-α4	Serotec^f^ (44H6)	Part of β_1_ integrin adhesion molecule complex
CD54	Intercellular adhesion molecule-1	Southern Biotechnology^g^ (15.2)	Adhesion molecule
CD62L	L-selectin	Coulter (DREG56) or Pharmigen (DREG56)	Adhesion molecule

a San Jose, CA.

b Plymouth Meeting, PA.

c Miami, FL.

d Bayport, MN.

e Measured in UP50 and UPASTHMA only.

f Raleigh, NC.

g Birmingham, AL.

**Table 3:** Exposure parameters (mean ± SD).

	UPREST	UPDOSE	UPDOSE	UP50	UPASTHMA
Nominal particle mass (μg/m^3^)	10	10	25	50	10
Measured particle mass (μg/m^3^)	10.00 ± 2.14	13.87 ± 4.02	28.46 ± 5.13	49.97 ± 3.88	11.08 ± 3.11
Particle number (× 10^6^ particles/cm^3^)	1.88 ± 0.09	2.04 ± 0.07	6.96 ± 0.10	10.79 ± 1.66	2.20 ± 0.10
CMD (nm)	27.3 ± 2.5	25.2 ± 1.7	26.5 ± 1.5	27.9 ± 2.2	23.1 ± 1.6
GSD	1.62 ± 0.02	1.60 ± 0.02	1.60 ± 0.02	1.65 ± 0.02	1.64 ± 0.01

Abbreviations: CMD, count median diameter; GSD, geometric standard deviation.

**Table 4:** Adhesion molecule expression on monocytes and PMNs, UPDOSE protocol (mean ± SE, MESF).

	Exposure (μg/m^3^)	Baseline	0 hr	3.5 hr	21 hr	ANOVA
Monocytes						
CD11a	Air	64,429 ± 2,072	62,483 ± 2,140	62,571 ± 1,689	65,682 ± 2,435	
	UFP 10	63,818 ± 4,109	59,900 ± 2,493	59,190 ± 3,063	65,249 ± 2,518	
	UFP 25	62,835 ± 2,644	56,207 ± 5,436	59,635 ± 2,404	63,008 ± 2,126	
CD11b	Air	19,034 ± 986	19,497 ± 997	21,076 ± 1,653	20,901 ± 1,912	
	UFP 10	17,632 ± 990	17,287 ± 1,171	18,335 ± 1,501	19,391 ± 1,185	
	UFP 25	19,056 ± 1,214	17,769 ± 922	22,059 ± 4,697	22,669 ± 3,357	
CD49d	Air	14,222 ± 1,000	13,562 ± 854	13,717 ± 880	13,989 ± 964	
	UFP 10	13,634 ± 1,029	12,587 ± 694	12,946 ± 706	13,059 ± 797	
	UFP 25	13,590 ± 839	12,779 ± 574	12,372 ± 683	13,542 ± 935	
CD54	Air	12,188 ± 319	13,096 ± 519	13,908 ± 645	13,307 ± 823	Exposure
	UFP 10	12,541 ± 469	12,470 ± 583	12,855 ± 592	13,110 ± 781	*p* = 0.001
	UFP 25	13,717 ± 686	12,591 ± 584	13,533 ± 856	14,482 ± 991	
CD62L	Air	43,970 ± 3,212	34,937 ± 3,519	37,600 ± 3,391	37,399 ± 3,716	Exposure × sex
	UFP 10	38,953 ± 3,465	30,281 ± 2,510	32,409 ± 1,719	36,356 ± 3,207	*p* = 0.006
	UFP 25	41,357 ± 4,453	33,134 ± 2,940	34,676 ± 3,234	39,168 ± 4,196	
PMNs						
CD11a	Air	28,637 ± 1,073	28,613 ± 1,228	28,793 ± 1,183	28,867 ± 1,503	
	UFP 10	29,124 ± 1,073	26,216 ± 1,160	26,260 ± 985	27,620 ± 923	
	UFP 25	28,444 ± 1,397	27,939 ± 1,151	27,817 ± 1,137	27,157 ± 1,411	
CD11b	Air	18,467 ± 1,117	18,837 ± 1,223	21,427 ± 3,186	21,189 ± 2,383	
	UFP 10	16,728 ± 907	15,997 ± 1,175	16,049 ± 1,112	21,169 ± 2,394	
	UFP 25	19,778 ± 2,671	15,671 ± 1,179	20,461 ± 3,457	18,653 ± 1,760	
CD49d	Air	7,422 ± 593	6,572 ± 542	6,404 ± 498	6,098 ± 686	Exposure × sex
	UFP 10	7,007 ± 561	6,172 ± 559	6,173 ± 423	6,340 ± 650	*p* = 0.007
	UFP 25	6,681 ± 465	6,031 ± 442	5,677 ± 446	5,925 ± 470	
CD54	Air	4,792 ± 279	4,500 ± 280	4,586 ± 246	4,457 ± 243	
	UFP 10	4,953 ± 271	4,292 ± 242	4,608 ± 424	4,435 ± 213	
	UFP 25	4,771 ± 321	4,084 ± 216	4,122 ± 215	4,417 ± 230	
CD62L	Air	66,179 ± 3,910	59,419 ± 4,413	64,867 ± 4,303	59,671 ± 5,970	
	UFP 10	60,976 ± 4,340	57,202 ± 4,515	56,621 ± 4,636	60,626 ± 4,180	
	UFP 25	66,145 ± 4,231	60,044 ± 5,434	59,625 ± 4,296	61,184 ± 4,054	

**Table 5:** Adhesion molecule expression on monocytes and PMNs, UP50 protocol (mean ± SE, MESF).

	Exposure	Baseline	0 hr	3.5 hr	21 hr	45 hr	ANOVA
Monocytes							
CD11a	Air	65,882 ± 3,277	66,463 ± 2,934	65,658 ± 2,963	69,888 ± 2,853	71,292 ± 2,885	
	UFP	69,090 ± 3,146	68,680 ± 2,935	66,222 ± 2,696	69,813 ± 2,835	71,773 ± 3,132	
CD11b	Air	16,840 ± 899	20,104 ± 905	19,938 ± 835	18,728 ± 1,092	18,364 ± 993	
	UFP	18,365 ± 1,153	19,733 ± 1,206	18,531 ± 952	18,389 ± 932	18,369 ± 815	
CD18	Air	62,675 ± 2,948	68,897 ± 2,942	67,872 ± 2,780	68,661 ± 2,749	68,963 ± 3,187	Exposure
	UFP	67,246 ± 2,751	67,175 ± 2,582	66,277 ± 2,488	67,307 ± 2,768	68,754 ± 3,052	*p* = 0.0002
CD49d	Air	16,334 ± 939	16,588 ± 859	17,371 ± 954	16,951 ± 9,571	17,126 ± 1,079	
	UFP	16,643 ± 938	16,445 ± 874	17,182 ± 965	17,282 ± 909	17,484 ± 1,167	
CD54	Air	9,637 ± 1,431	10,654 ± 1,668	11,198 ± 1,728	9,969 ± 1,639	9,827 ± 1,687	Exposure × sex
	UFP	10,526 ± 1,715	11,095 ± 1,782	10,889 ± 1,871	10,352 ± 1,791	10,339 ± 1,811	*p* = 0.025
CD62L	Air	58,551 ± 3,188	50,197 ± 3,410	48,580 ± 3,027	9,699 ± 1,557	59,189 ± 2,271	
	UFP	57,666 ± 3,519	49,307 ± 3,261	50,241 ± 2,848	56,880 ± 3,515	58,283 ± 3,020	
PMNs							
CD11a	Air	30,921 ± 851	30,934 ± 862	31,339 ± 960	31,683 ± 944	31,712 ± 937	Exposure × time
	UFP	31,569 ± 1,014	32,158 ± 1,055	31,652 ± 912	31,751 ± 927	32,130 ± 921	*p* = 0.037
CD11b	Air	16,406 ± 628	18,053 ± 934	17,262 ± 678	17,355 ± 869	17,525 ± 848	
	UFP	16,678 ± 830	19,155 ± 1,953	17,076 ± 777	18,014 ± 713	17,545 ± 694	
CD18	Air	34,919 ± 1,335	36,961 ± 1,352	36,486 ± 1,286	35,907 ± 1,226	35,868 ± 1,450	Exposure
	UFP	36,010 ± 1,032	37,687 ± 1,810	36,255 ± 1,060	35,316 ± 983	35,682 ± 1,087	*p* = 0.023
CD49d	Air	6,455 ± 412	6,345 ± 264	6,399 ± 279	6,145 ± 204	6,070 ± 203	
	UFP	6,186 ± 335	6,252 ± 330	6,362 ± 340	6,284 ± 305	6,114 ± 258	
CD54	Air	8,182 ± 584	8,339 ± 484	8,973 ± 552	8,114 ± 415	8,072 ± 383	
	UFP	8,524 ± 427	9,071 ± 545	8,668 ± 458	8,501 ± 402	8,446 ± 389	
CD62L	Air	87,437 ± 4,510	88,596 ± 3,485	88,617 ± 4,056	87,244 ± 3,362	89,489 ± 2,648	
	UFP	92,053 ± 4,760	89,783 ± 4,262	90,736 ± 4,227	89,363 ± 3,898	94,055 ± 4,598	

**Table 6:** Adhesion molecule expression on monocytes and PMNs, UPASTHMA protocol (mean ± SE, MESF).

	Exposure	Baseline	0 hr	3.5 hr	21 hr	45 hr	ANOVA
Monocytes							
CD11a	Air	21,179 ± 4,120	20,442 ± 3,989	19,336 ± 4,042	21,126 ± 5,569	21,407 ± 5,550	
	UFP	32,102 ± 7,076	30,277 ± 6,791	29,592 ± 6,630	30,468 ± 6,809	29,751 ± 6,640	
CD11b	Air	25,022 ± 2,822	31,626 ± 5,969	26,553 ± 3,319	26,345 ± 3,456	27,703 ± 3,228	Exposure
	UFP	26,958 ± 4,112	25,452 ± 4,611	25,742 ± 4,241	24,498 ± 4,199	25,814 ± 3,502	*p* = 0.029
CD18	Air	85,586 ± 6,773	87,234 ± 8,882	82,899 ± 6,465	82,697 ± 7,370	85,455 ± 7,819	
	UFP	84,999 ± 7,252	81,131 ± 7,931	81,297 ± 9,950	82,028 ± 6,767	77,346 ± 7,334	
CD49d	Air	17,172 ± 731	16,739 ± 925	16,013 ± 616	16,627 ± 837	16,856 ± 771	
	UFP	18,378 ± 865	16,967 ± 873	17,138 ± 919	17,715 ± 877	17,327 ± 879	
CD54	Air	19,102 ± 1,386	19,432 ± 1,430	18,285 ± 1,248	19,043 ± 1,410	19,281 ± 1,319	
	UFP	20,673 ± 2,009	20,438 ± 2,088	19,861 ± 1,934	20,014 ± 1,853	19,284 ± 1,491	
CD62L	Air	45,571 ± 2,571	39,446 ± 2,652	41,214 ± 2,703	45,100 ± 2,847	44,329 ± 2,870	
	UFP	51,939 ± 5,305	43,483 ± 4,955	42,198 ± 3,954	46,105 ± 4,023	45,608 ± 4,271	
PMNs							
CD11a	Air	10,540 ± 1,775	10,010 ± 1,771	10,107 ± 1,837	10,986 ± 2,830	11,199 ± 2,953	
	UFP	14,562 ± 2,749	14,161 ± 2,679	13,790 ± 2,780	13,765 ± 2,727	13,710 ± 2,652	
CD11b	Air	24,078 ± 2,783	26,353 ± 3,578	25,211 ± 2,533	25,199 ± 2,072	30,893 ± 4,350	
	UFP	23,819 ± 2,343	22,792 ± 3,224	25,376 ± 2,984	22,085 ± 2,479	22,781 ± 1,886	
CD18	Air	48,861 ± 3,054	47,564 ± 3,026	45,449 ± 2,457	45,303 ± 2,719	50,312 ± 5,429	
	UFP	46,982 ± 2,925	44,465 ± 2,676	43,512 ± 3,174	44,599 ± 2,862	43,470 ± 3,006	
CD49d	Air	5,342 ± 211	5,122 ± 228	5,090 ± 162	4,805 ± 248	4,923 ± 185	
	UFP	5,499 ± 315	4,964 ± 212	4,887 ± 210	4,783 ± 234	4,950 ± 241	
CD54	Air	5,631 ± 230	5,348 ± 236	5,234 ± 222	5,433 ± 277	5,635 ± 239	Exposure × time
	UFP	6,262 ± 451	5,759 ± 453	5,604 ± 458	5,535 ± 399	5,660 ± 398	*p* = 0.031
CD62L	Air	78,859 ± 3,812	69,825 ± 3,978	71,796 ± 3,691	72,829 ± 4,711	72,429 ± 4,184	Exposure × sex
	UFP	79,315 ± 6,332	75,646 ± 6,405	70,468 ± 4,961	74,971 ± 5,500	74,541 ± 5,925	*p* = 0.011

**Table 7:** Blood leukocyte marker expression at baseline that differed between asthmatic and healthy subjects (mean ± SE, MESF).

	Healthy^a^	Asthma	*p*-Value
Lymphocytes			
CD11a	41,710 ± 1,844	14,575 ± 4,161	< 0.001
CD11b	1,460 ± 67	1,784 ± 107	0.017
CD49d	8,168 ± 335	10,486 ± 324	< 0.001
CD54	2,381 ± 69	2,964 ± 155	0.003
Monocytes			
CD11a	64,155 ± 4,041	26,220 ± 5,260	< 0.001
CD11b	17,944 ± 915	25,047 ± 2,751	0.025
CD49d	13,556 ± 915	17,089 ± 642	0.005
CD54	12,314 ± 401	17,942 ± 1,065	< 0.001
PMNs			
CD11a	28,358 ± 904	12,753 ± 2,276	< 0.001
CD11b	16,868 ± 1,055	24,178 ± 2,705	0.021
CD49d	7,189 ± 545	5,292 ± 282	0.007
CD62L	63,591 ± 4,614	80,656 ± 5,954	0.032

a Includes subjects from UPREST and UPDOSE (*n* = 24). Source of some immunofluorescence markers differed for UP50, resulting in changes in baseline values, so these healthy subjects were not included.

**Table 8:** Summary of UFP exposure effects.

Protocol	Adhesion molecules	Lymphocyte subsets and activation	Leukocyte counts
UPREST (*n* = 12)	No convincing effects (see text)	No effects	No effects
UPDOSE (*n* = 12)	Decreased monocyte CD54 Decreased PMN CD49d (males)	Increased CD25^+^ T cells (females)	Decreased monocytes and basophils (females)
UP50 (*n* = 16)	Decreased monocyte CD18 and CD54 (males)	Increased CD25^+^ T cells	Decreased eosinophils
	Decreased PMN CD18 and increased CD11a		
UPASTHMA (*n* = 16)	Decreased monocyte CD11b Decreased PMN CD54 and increased CD62L (males) Decreased eosinophil CD11b	Decreased CD4^+^ T cells and basophils	Decreased eosinophils
